# Uncovering the Hidden Burden of Pharmaceutical Poisoning in High-Income and Low-Middle-Income Countries: A Scoping Review

**DOI:** 10.3390/pharmacy11060184

**Published:** 2023-11-24

**Authors:** Claire Cowans, Anya Love, Balamurugan Tangiisuran, Sabrina Anne Jacob

**Affiliations:** 1 Strathclyde Institute of Pharmacy and Biomedical Sciences, Glasgow G4 0RE, UK; claire.cowans@wales.nhs.uk (C.C.); anya.love2@nhs.scot (A.L.); 2 School of Pharmaceutical Sciences, Universiti Sains Malaysia, Gelugor 11800, Penang, Malaysia; bala@usm.my; 3 Jeffrey Cheah School of Medicine and Health Sciences, Monash University Malaysia, Jalan Lagoon Selatan, Bandar Sunway 47500, Selangor, Malaysia

**Keywords:** pharmaceutical, poisoning, low-and-middle-income countries, high-income countries, review

## Abstract

Pharmaceutical poisoning is a significant global public health concern, causing approximately 190,000 deaths annually. This scoping review aims to comprehensively map the available literature on pharmaceutical poisoning and compare patterns between high-income countries (HICs) and low-middle-income countries (LMICs). A systematic search was performed across the following databases: Embase, PubMed, Cochrane Database of Systematic Reviews, Cochrane Central Register of Controlled Trials, and CINAHL. Studies included were from 1 January 2011 to 31 December 2020, in English, with full text available. Seventy-nine articles were included in the study; 21 were from LMICs and 58 were from HICs. Toxic exposure was largely intentional (77%) in LMICs and accidental (68%) in HICs. Drugs acting on the nervous system were responsible for 95% of toxicities worldwide with analgesics accounting for the largest subtherapeutic group in both LMICs (40%) and HICs (58%). Notable statistics were that HICs accounted for 99% of opioid overdoses, and LMICs accounted for 19% of anti-epileptic-induced toxicities. Overall, the medical outcomes due to poisonings were generally worse in LMICs. The review provides possible interventions to target specific geographic locations, based on the trends identified, to reduce the burden worldwide. Many gaps within the literature were recognised, calling for more robust analytical research.

## 1. Introduction

Toxic exposure to medicines remains a significant, under-recognised global public health concern. The World Drug Report estimates that pharmaceutical poisoning causes 190,000 fatalities annually [1]; with non-fatal poisoning 20–30 times more prevalent and often causing long-term morbidities [2]. The most prevalent long-term conditions are respiratory, renal or hepatic failure, cognitive impairment, and hypoxic brain injury, depending on the drug(s) involved [3]. This severely reduces patients’ quality of life and puts a strain on healthcare services and society worldwide. 

Drug-induced toxicities require immediate action by emergency medicine and national toxicology centres. This creates immense pressure on healthcare systems, apparent from observing hospital admissions alone. In the United Kingdom (UK), approximately 100,000 patients present to emergency departments annually due to drug poisoning, which in turn is responsible for 10% of general ward admissions [4]. Likewise, almost 75% of drug overdose cases in Japan require ambulance services, which account for 15% of intensive care unit (ICU) admissions [5]. Similar trends have been observed worldwide highlighting the burden on emergency services, consuming valuable resources and delaying care for other life-threatening emergencies [5].

Pharmaceutical poisoning can be categorised as intentional (deliberate) or unintentional (accidental). The latter ranks fifth in European injury-related mortality, with the highest rates in Lithuania, Ireland, Estonia, Romania and Latvia [6]. Most unintentional drug poisoning cases occur in children under five, from having a natural curiosity to explore unfamiliar objects and failing to recognise the associated dangers due to their developing cognitive function [7]. Such incidents are most common within a household setting where 10–20% of child exposures are due to their grandparent’s medicines being easily accessible [8]. Many co-morbidities in the elderly require multiple medications, which accumulate in homes [9]. Patients often self-manage their medicines using blister packs, removing the drugs from their original, child-resistant, packaging—consequently increasing the risk of accidental consumption and overdose [10]. Therapeutic errors can also cause unintentional toxicity as well accidental consumption. Such errors are often caused by dosing errors, especially for high-risk medications with a narrow therapeutic index [6].

Alarmingly, most drug toxicity cases are due to intentional self-harm. These intentional exposures occur in countries worldwide regardless of income status, often due to distressing life events, poverty, and psychiatric illnesses, with the highest rates among adults aged 33–44 [11,12]. In 2016, over one billion people worldwide were diagnosed with a mental health condition, 20% of whom were children or adolescents [13,14]. Many of these patients are prescribed drugs to help manage their conditions, highlighting the magnitude of the population’s vulnerability and exposure to medicines with potential toxicities. Over 60% of drug poisoning suicides in Asia are from people with psychiatric conditions, highlighting the correlation between mental illness and pharmaceutical poisoning [1,15,16]. Furthermore, overdosing with prescribed and over-the-counter medicines accounts for 79% of UK emergency department presentations due to self-harm [3,16,17]. 

Opioids are the major cause of drug-induced toxicity globally [18]. In the Global Burden of Disease Study 2017, 109,500 people died from opioid usage, including prescription, synthetic, and illegal opioids [17,19]. Due to relaxed drug classification and illicit marketplaces availability, the USA has an opioid pandemic. Indeed, over the past 20 years, the USA opioid pandemic has quadrupled in mortality [20]. Similarly, opioids are the main driver of fatal overdoses in Europe, responsible for approximately eight out of 10 drug-induced deaths [21]. The UK and Germany, in particular, account for almost half (47%) of all opioid overdose mortalities in Europe [22]. 

Socioeconomic marginalisation and cultural differences affect pharmaceutical poisoning regionally. In high-income countries (HICs), medicines are responsible for over 50% of all poisonings [23]. In contrast, in low-middle-income countries (LMICs) such as Ethiopia, India, and Sri Lanka, household products, organophosphates, and pesticides are the major contributors to poisonings, with pharmaceuticals accounting for as little as 10% of toxicities [24]. Because a substantial portion of the population in these places rely on agriculture for money or work, such products are readily available and commonly misused [25,26]. However, drug-overdose mortality is still estimated to be four times higher in LMICs compared to HICs [27]. These inconsistencies are caused by differences in global medicine regulation authority. The lack of regulatory bodies in many LMICs leads to poor access to quality medicines, a higher risk of exposure to falsified drugs, poor prescribing policies, and lenient laws surrounding over-the-counter medicines, where 60% of drugs in developing countries are prescribed or sold inappropriately [28,29,30], contributing to global drug-poisoning disparities.

While extensive literature has been published on pharmaceutical poisoning in specific countries, no efforts have been made to collate this data and analyse trends globally. This would provide an overall evaluation of the key themes of pharmaceutical poisoning and highlight the impact of a country’s income level on such patterns. 

This scoping review aims to identify the available literature and compare the patterns of pharmaceutical poisoning between LMICs and HICs, specifically focusing on the reason(s) for exposure, the drug(s) responsible, and the medical outcome(s). All drug poisoning cases are avoidable, so understanding patterns can assist in developing preventative strategies and prioritising geographical areas most in need to target such campaigns.

## 2. Materials and Methods

This study was conducted according to the Preferred Reporting Items for Systematic Reviews and Meta-Analyses Extension for Scoping Reviews (PRISMA-ScR) (Supplementary Table S1) [31]. 

### 2.1. Data Sources and Search Strategy 

A comprehensive, systematic search was completed using five electronic databases: Embase, PubMed, Cochrane Database of Systematic Reviews, Cochrane Central Register of Controlled Trials and CINAHL. In order to form the search strategy, the study objectives were translated into search terms to ensure all relevant articles were captured. This was achieved by completing an initial search on PubMed to identify relevant papers on the topic. Papers were analysed for keywords used in the title and abstract to describe the subject area. The keywords identified formed the search strategy that was used to search the five databases, available in Appendix A. The terms were a combination of words to describe ‘poisoning’ and ‘pharmaceuticals’ as displayed in Table 1. The search results were restricted to articles published from 1 January 2011 to 31 December 2020. In 1997, the World Health Organization (WHO) issued recommendations for poison control. The guidelines highlighted the importance of standardizing poisoning diagnostic and treatment data collection, toxicovigilance, and poison prevention initiatives [32]. The revised version of ‘Guidelines for Establishing a Poison Centre’ followed in early 2021 [33]. We analyzed a decade-long trend using a 2011–2020 criterion prior to the publication of the updated version. A further manual search on Google Scholar was completed to identify any grey literature.

Search results were imported into Endnote 20 (Thomson Reuters, New York, NY, USA) where they were grouped according to the database they were sourced from. Each group was then uploaded to Covidence for screening where duplicates of articles were removed. Two reviewers (CC and AL) independently screened all titles and abstracts of the remaining articles. Bibliographies of relevant studies were also checked for additional publications. Full-texts of potentially relevant studies were then reviewed independently by both reviewers to confirm eligibility according to the inclusion and exclusion criteria. Any discrepancies were discussed between both reviewers, and if a consensus could not be reached, the lead researcher (SAJ) was consulted. 

### 2.2. Study Selection

Studies were included if they fulfilled the following criteria: (1) the study reported on poisoning due to pharmaceuticals; (2) the published date was between 1 January 2011–31 December 2020; (3) full texts and abstracts were available in English; (4) the country where the study was conducted was stated; and (5) the article stated both the reason(s) (e.g., accidental or intentional) and outcome(s) (e.g., length of hospital stay, morbidity or mortality) of the poisoning. Studies were excluded from this review if: (1) they reported on poisoning due to toxins other than medicines (e.g., household products, pesticides etc.) or there was no separation of results between different toxins; and (2) they reported on illicit drug poisoning or did not separate results between medicinal drugs and illicit substances. Reviews, systemic reviews, scoping reviews, meta-analyses, in vitro and in vivo studies, animal studies, conference abstracts or proceedings, reports, letters to the editor, and comments were also excluded.

### 2.3. Data Extraction and Synthesis 

A data-charting form was developed to capsulate the variables required to be extracted from the included studies. This was trialed on five articles to ensure the relevant data was easily charted and the form was altered accordingly. The following data were extracted and tabulated from included studies: (1) author and year of publication; (2) study design and objectives; (3) location of the study; (4) sample size; (5) demographic characteristics including age and gender; (6) reason for exposure; (7) drug(s) responsible for toxicity; and (8) patient-related outcome(s) of poisoning. Extracted information from studies were grouped according to the income status of the country where the study was conducted. Income status was categorised into ‘LMIC’ and ‘HIC’ with reference to the World Bank Country Classifications by Income Level 2021–2022, defined by gross national income per capita [34]. 

In order to aid identification of the common drug classifications responsible for the poisoning, the Anatomic Therapeutic Chemical (ATC) and Defined Daily Dose (DDD) (ATC/DDD) Toolkit was used to classify drugs into the organ or biological system they target [35]. Some publications present the outcome of the drug poisoning according to the Poisoning Severity Score (PSS), which ranks the severity of the toxicity. The system scores poison outcomes as (0) no effect (patient is asymptomatic); (1) minor effect (mild symptoms); (2) moderate effect (prolonged symptoms); (3) severe (life-threatening symptoms with significant residual disability or disfigurement), or (4) fatal [36]. Finally, age categories were defined and categorised using the WHO definition, which states that a child is under the age of 18 and an adult is 18 years or over. These categories were used when analysing patient demographic trends and the effect of age on pharmaceutical poisoning [37]. Key patterns identified from the extracted data were summarised narratively with the aid of tables and charts into key categories.

## 3. Results

### 3.1. Characteristics of Included Studies 

**Figure 1:**
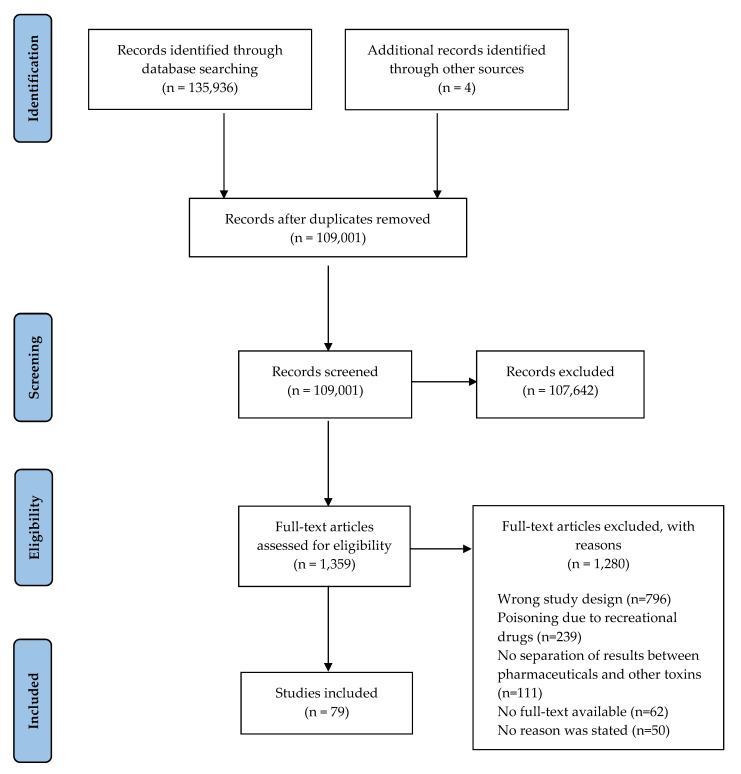
PRISMA-ScR flowchart of study selection.

The initial search identified 135,936 publications, with four additional studies identified during a manual search on Google Scholar. After screening titles and abstracts, 1359 studies met the inclusion criteria. Full texts of the 1359 studies were assessed for eligibility, where a further 1280 were excluded for the following reasons: wrong study design (n = 796), no separation of results between pharmaceutical drugs and illicit substances (n = 239), no separation of results between pharmaceutical poison and other types of poisoning (n = 111), no full-text available (n = 62), and the study failed to state the reason(s) for poisoning (n = 50) or outcome(s) (n = 22). This resulted in 79 studies being included in the data synthesis of this scoping review, as summarised in the PRISMA-ScR diagram (Figure 1). 

Of the included studies, eight were prospective studies: one survey, five cross-sectional studies, one cohort study, and one observational follow-up. The remaining 71 were retrospective studies: 63 cohort and eight cross-sectional studies. Tables presenting a summary of the study characteristics included in this review can be found in Table A1 and Table A2 in Appendix B. 

### 3.2. Overview

The collective sample size of participants was 1,660,165 (HICs: 1,653,519; LMICs: 6646), with ages ranging from one month old to 100 years old. Of the total study group where gender was stated, 51.1% were female (n = 694,234). Twenty-one of the studies (27%) were conducted in LMICs: one each in Algeria, Argentina, Jordan, Morocco, Romania, South Africa, and Sri Lanka; two in India, three in Turkey, and nine in Iran. Fifty-eight studies (73%) were conducted in HICs: 27 in the USA, four in Switzerland, three each in Canada, France, and Denmark; two each in Australia, Israel, Japan, Poland, Finland, and Saudi Arabia, and one study each in the Czech Republic, Republic of Ireland, New Zealand, Singapore, Taiwan, and the UK (Table 2). 

### 3.3. Trends 

The results of the scoping review are presented in three broad categories: (i) the reason behind the exposure to drug poisoning, (ii) the pharmaceutical agent responsible for toxicity, and (iii) the medical outcomes of poisonings.

#### 3.3.1. Reason behind Toxic Exposure 

The reason for poisoning was classified into two broad categories: intentional or unintentional (accidental). Of the overall sample size, 95% (n = 1,577,159) stated the known reason for being exposed to the drugs at toxic levels with the remaining 5% unknown. For studies that were set in LMICs, 76.2% of exposures were intentional (n = 4809). Further reasons for intentional poisoning were stated for 67% (n = 3216), with attempted suicide accounting for 91.8% (n = 2952), self-harm for 5.3% (n = 172), relationship conflicts noted for 2.2% (n = 72) and homicide for 0.6% (n = 20). For the 23.8% (n = 1503) of patients that were exposed to drug poisoning accidently, detailed reasons were given for 15% (n = 232) and included 31.5% due to careless storage (n = 72), 18.1% due to parental mistakes (n = 42), 18.5% due to therapeutic errors (n = 43), and 31.9% due to ingestion by children while playing (n = 74). 

**Figure 2:**
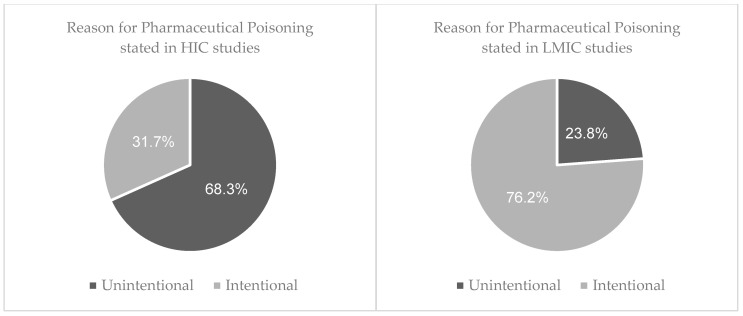
Comparing the reason behind pharmaceutical poisoning between LMIC and HIC’s.

In HICs, 31.7% (n = 499,332) of exposures were intentional. Additional explanations for intentional exposure were given for 5% (n = 25,828); with 92.3% stating attempted suicide (n = 23,829), 6.8% as misuse (n = 1763) and the remaining 0.9% stating abuse of the drug (n = 236). Unintentional poisoning was reported in 68.3% (n = 1,075,873) of cases. Further explanations for accidental exposures included 94.7% as therapeutic errors (n = 508,402), 1.3% as adverse drug reactions (n = 6847), and 4% due to one or more products containing the same active ingredient being consumed (n = 21,361). (Figure 2).

A common trend seen over the included studies was that the intent behind the pharmaceutical poisoning varied depending on age. Fifteen of the studies reported on pharmaceutical poisoning in children, of which 70.2% were exposed accidently (n = 76,398) [42,49,61,67,75,76,78,82,86,91,92,94,95,96,97]. Five studies had separated results for adult exposure where 80.9% of exposures were intentional (n = 725) [73,78,94,96,98].

#### 3.3.2. Types of Pharmaceuticals Responsible for Poisoning 

Using the Anatomical Therapeutic Chemical (ATC) Classification toolkit via the WHO, causative drugs responsible for poisoning were divided into the 1st level classification, which has 14 main anatomical or pharmacological groups (Table 3). Of the studies that specifically mentioned the drug(s) responsible for the poisoning, 94.7% (n = 1,368,876) were pharmaceuticals categorised under ‘Nervous System’: 54% (n = 3069) and 95% (n = 1,365,780) in LMICs and HICs respectively. 

Looking more closely at the ‘Nervous System’ identified the therapeutic subgroups most commonly responsible (Table 4). In LMICs, 40% (n = 1236) of the central nervous system (CNS)-acting medicines exposed were analgesics, of which 39% were paracetamol, and 31% were from exposure to prescription opioids. In HICs, analgesics accounted for close to 60% of drugs acting on the nervous system, of which 73% (n = 567,925) were prescription opioids and 25% (n = 198,282) paracetamol. Psychoanaleptics (antidepressants, psychostimulants, and anti-dementia drugs) accounted for more than 30% (n = 461,019) of CNS agents. When looking at the global exposures to nervous system agents, LMICs were responsible for less than 1% of toxicities from analgesics, psycholeptics, and psychoanaleptics; 4% of drugs used in opioid dependence, and 19% of toxicities due to antiepileptics.

#### 3.3.3. Outcome of Pharmaceutical Poisoning 

Of those hospitalised, 85% were in LMICs (n = 5668) and 20% in HICs (n = 327,439). Across all studies, the average time hospitalised varied from 17.1 h to 13.9 days ranging from 5 h to 91 days [38,54,73]. Less than 1% (n = 11,237) were admitted to the ICU due to poison exposure, where admissions accounted for 10% of the LMIC population outcomes (n = 666) and less than 1% of HIC outcomes (n = 10,571). The most common medical outcomes were all observed in less than 1% of the total study size and included acute kidney injury (n = 9126), organ failure (n = 2765), coma (n = 6776), respiratory depression (n = 6839) and seizures (n = 418). 

Nine out of the 79 studies utilised the PSS as a measure of medical outcome [46,50,87,95,96,97,99,100,101]. One was set in Jordan (LMIC) and the remaining eight reported on outcomes from HICs. In the Jordan study, 40% were asymptomatic (n = 363), 39% had mild symptoms (n = 355), 17% were moderate (n = 150) and 4% severe (n = 32). For those reporting using the PSS in HICs (n = 285,481), 56% were asymptomatic (n = 161,269), 32% experienced minor symptoms (n = 90,819), 11% had moderate effects (n = 30,035), 1% were severe (n = 3075) and less than 1% of the poisonings were classified as fatal (n = 283).

Overall, 20,314 deaths were recorded across all included studies. In LMICs, 2% of the pharmaceutical poisoning outcome was death (n = 137), while in HIC 1.2% deaths (n = 20,177) were reported. A key trend observed was the exposure to toxic levels of CNS-acting drugs causing mortality. Eight articles reported deaths as the sole outcome of drug poisoning. Over the eight articles, the collative sample size was 16,175. The five major drug groups responsible for mortality were opioids (47%), anxiolytics (14%), antidepressants (12%), anti-epileptics (5%) and methadone for opioid substitution therapy (4%) [58,60,65,70,89,90,102,103].

## 4. Discussion

After synthesising the data from the 79 papers that met the inclusion criteria, specific trends between economically developed and developing countries were identified, and research gaps were recognised. 

### 4.1. Reason behind Toxic Exposure 

The disparity in reasons for pharmaceutical poisoning between LMICs and HICs was remarkable. Over 75% of LMICs’ exposures were deliberate self-poisonings, with 92% further stating overdose with the intent of suicide. Previous literature has recognised the gravity of the issue in the developing world, with eight of the top ten countries with the highest suicide rates being LMICs [104]. In contrast, accidental exposure to pharmaceuticals accounted for 68% of toxicities in HICs, with over 94% of these due to therapeutic errors, including administration errors, consuming multiple medicines with the same active ingredient, adverse drug reactions, and poor storage leading to child exposure. This finding may be due to more efficient error reporting and surveillance systems in developed countries [105]. 

With regard to the effect of age on poisoning, the results reaffirmed that child toxicities are predominantly unintentional, with adults mostly intentional in both LMICs and HICs [7]. The disparity in the causes of pharmaceutical poisoning between LMIC and HIC is likely attributable to a number of socioeconomic factors, including the availability of healthcare resources, poverty, access to treatment and support services, cultural attitudes towards mental health, and other socioeconomic factors. Higher rates of intentional self-poisoning with suicidal intent in LMICs reflect a lack of access to mental health resources and support, poverty and bad living conditions, or a cultural stigma associated with seeking assistance for mental health difficulties. In contrast, accidental poisonings may be more widespread in HICs due to higher access and availability of pharmaceutical medications, and a lack of knowledge or education regarding their proper use and potential risks [106,107,108].

### 4.2. Types of Pharmaceuticals Responsible for Poisoning 

The overwhelming majority (94.7%) of pharmaceutical toxicities worldwide were from drugs acting on the nervous system, with analgesics accounting for the largest sub-group responsible. Opioids were responsible for most analgesic exposures, with the problem largely residing in HICs, likely due to their accessibility in these areas being far greater than for LMICs, where a considerable lack of pain relief medications is available [109]. Indeed, in a Lancet Commission Report, it was reported that only 0.1 metric tonne of morphine-equivalent opioids are delivered to LMICs, from almost 300 metric tonnes [110]. Furthermore, overprescribing and long-term use of opioids are considered the root cause of toxicities in HICs due to risks of dependence, often leading to misuse and overconsumption [111]. Medicines used in opioid substitution treatment were also commonly responsible for the poisoning, perhaps due to the vulnerability of patients receiving such treatment and the risk of co-ingesting opiates along with substitution therapy. 

Findings from this review also revealed that poisoning due to psychoanaleptics accounted for the second largest subtherapeutic group in HICs, while psycholeptics were the second largest in LMICs. Similar results have previously been reported where analgesics, psycholeptics (mostly benzodiazepines), and pschoanaleptics (particularly antidepressants) were the groups largely responsible for intoxication [112]. The results also matched previous findings where toxicity due to a combination of drugs was common in LMICs and HICs due to the risks of drug-drug interactions. Despite these three subgroups accounting for most pharmaceutical toxicities worldwide, LMICs were responsible for less than 1% of these poisonings meaning the problem significantly exists within HICs. However, a subgroup where LMICs were particularly accountable for the global burden was exposure to antiepileptics, where almost 20% of toxicities occurred in these developing countries. Part of the explanation may be that 85% of epileptic patients reside in LMICs [113]. Furthermore, antiepileptics are approved for a number of indications besides the treatment of epilepsy, including neuropathic pain and mood stabilisation, common conditions prevalent in these areas and two major groups vulnerable to intentional overdose and suicide ideation [113,114]. Additionally, access to anticonvulsants is far more attainable than analgesics in these deprived countries, particularly first-generation anticonvulsants, which are notorious for their poor safety profile with a high risk of toxicity in comparison to second-generation agents [113].

### 4.3. The Outcome of Pharmaceutical Poisoning 

Analysing the outcome of drug-related poisoning, findings revealed that 85% and 20% of those exposed were in LMICs and HICs respectively, with the duration of hospital stay ranging from five hours to 91 days. Admissions to the ICU were over 10 times more common in the developing world, and fatality rates from overdose were almost twice as high compared to HICs. This can be explained by the intent affecting the outcome where there is a direct correlation between the dose consumed and a worse prognosis. Thus, mortalities are higher in LMICs as far larger quantities are likely to be consumed when the exposure was intentional. Furthermore, the disparities in healthcare resources are also responsible for poorer outcomes. Access to healthcare resources and poison information centres that advise on the management of poisoning is far scarcer in LMICs, leading to delayed treatment and interventions, increasing the exposure length and ultimately worsening the outcome [115]. For studies in this scoping review that reported according to the PSS, most outcomes were asymptomatic and mild in severity, and very little of the study population suffered from severe (life-threatening) or fatal effects. Therefore, findings reveal that pharmaceutical poisoning is associated with more short-term illnesses and morbidities than mortality. 

### 4.4. Future Research and Recommendations

When considering the geographical location of included studies, an uneven distribution between those conducted in LMICs and HICs was apparent. Despite over 85% of the world’s population residing in LMICs, there was a poor representation of the developing world, with 73% of the studies reporting on HICs [114]. Thus, obtaining an in-depth comparison of poisoning patterns between the economically developed and developing world was difficult. The low number of papers could be due to the exclusion of a large number of papers which did not separate between poisoning due to pharmaceuticals and other types of poisons. However, the lack of poison information centres partly justifies this, a major resource for collecting such data. According to the WHO, only 47% of countries have an established poison centre, with African, Eastern Mediterranean, and Western Pacific regions particularly lacking [116]. Therefore, it should be a public health priority for governments to invest funding into establishing and strengthening these centres. This would not only improve surveillance for future research but also guide managing drug-induced poisons, thus improving outcomes. 

Globally, the expenditure on mental health services is inadequate and is disproportionately worse in LMICs compared to HICs, with regard to the magnitude of the problem and the poisonings that arise from it [114]. It is estimated that globally, there is an average of 3.96 psychiatrists per 100,000 people. However, in developing countries such as Ethiopia, India, Nigeria, and Pakistan, those rates are 0.04, 0.301, 0.06, and 0.185, respectively [114]. Furthermore, within countries, there are large variations in access to mental health workers, with the majority often concentrated in urban areas meaning those living rurally have poor access and minimal support available [114]. There is an urgent need to train and employ more individuals in the mental health workforce to increase accessibility to non-pharmacological treatment. This would also limit the prescribing of psycholeptics and psychoanaleptics; two major drug classes highlighted in this scoping review to be responsible for toxicities. Furthermore, setting up referral schemes after patients are discharged from an intentional overdose to provide appropriate support would reduce the likelihood of reoccurrence. 

Due to the overwhelming impact of opioids on the burden of pharmaceutical poisoning, it is essential that improvements in national policies are made in the areas where opioid overdose is particularly problematic. There is an urgent need for improved legislation and policies with regard to the prescribing and duration of treatment with opioids as well as improved education on chronic pain management. Furthermore, better recognition of those requiring support from addiction services and increased access to the opioid-reversal agent naloxone would reduce the burden of opioid toxicities [117]. 

Those most vulnerable to opioid toxicity are often regular patients to pharmacies [118]; thus, having a supply of naloxone in every pharmacy and training staff on recognising the signs of an overdose and the protocol to follow would be immense in the prevention of life-threatening toxicities. That being said, it is important to consider the difficulties of implementing such strategies in both HICs and LMICs. In HICs such as the USA, there are relaxed policies and opioids are easily accessible [119]. While in LMICs, pharmacy services are reported to be lacking, with the drive being profit over patient care [120]. Furthermore, access to medicines is also limited [121], so having naloxone available in every community pharmacy may be logistically difficult. Perhaps having a national initiative scheme available to pharmacies to widen access to services within the community would help improve patient-centred care and reduce toxicities from occurring or refer those who present at risk in a reasonable time.

Many countries have yet to prioritise poisoning prevention strategies despite the severity of the issue. Public health campaigns focusing on increasing parental awareness of storing medicines in their original packaging and keeping them out of sight and reach of children are required to prevent the risk of confusing them for ‘sweets’ [6]. Many intentional poisonings are often impulsive; thus, limiting the accumulation of medicines stored in households by promoting safe disposal via pharmacies would be an effective strategy. Such campaigns could be promoted within healthcare settings and social media should be utilised to target large audiences [122]. 

Several research gaps were identified whilst conducting this scoping review. As discussed above, data available from LMICs were minimal, underlining the need for more robust analytical studies to reduce the disparity and underrepresentation of the developing world. In addition, research understanding the barriers to establishing poison information centres in LMICs and how these could be addressed would be valuable for enhancing the response to drug-induced toxicity in these regions despite the availability of multiple guidelines for establishing poison centres and other aspects of dealing with poisonings [33,123].

To address the disparity in patterns of pharmaceutical poisoning between LMICs and HICs, a less costly strategy of increasing awareness would be beneficial. This could be achieved by collecting and analysing the attitudes and competencies of healthcare professionals practising outside of hospitals towards managing drug-induced poisonings. This research could identify areas where further education and awareness of resources available, such as tox-based apps, would improve the triaging of patients and reduce unnecessary referrals from community settings to emergency departments.

As well as this, the findings revealed that hospitalisation and utilisation of emergency departments is a common outcome of drug-related poisoning despite many toxicities being asymptomatic or mild in severity. Thus, attempts to collect and analyse the attitudes and competencies of healthcare professionals practising in sectors beyond hospitals in advising and managing drug-induced poisons would be valuable. Additional personnel or qualified emergency physicians and the development of multidisciplinary teams in major hospitals in LMICs are also required to address the issue of pharmaceutical poisoning better. This will ensure that patients in emergency settings receive prompt and effective care and lessen the burden on the healthcare system. 

Generative AI technology has the ability to revolutionise how individuals obtain information about poisonings and seek medical care. By providing free and immediate access to information about various types of poisonings, their symptoms, and risk reduction strategies, this technology can assist individuals in determining if they or someone they know has been exposed to a harmful substance, thereby facilitating more targeted and effective treatment. There are limitations to chatbot AI technology despite its potential benefits. Challenges such as the quality and diversity of training data, the limitations of pre-programmed responses, and platform constraints can impact the accuracy and relevancy of the delivered information. It is crucial to use chatbot AI technology to complement professional medical advice, not as a replacement.

### 4.5. Strengths and Limitations 

This scoping review is the first attempt to collate the broad field of literature and identify patterns of pharmaceutical poisoning at a global level. A few limitations were noted. Firstly, only articles that were available in English were included, which likely limited the data available in non-native English-speaking countries. Secondly, a large number of initial studies were found during the search. Despite this ensuring all relevant papers were captured, it perhaps reflects that the search strategy was not specific enough to the study’s aims. We also excluded from the scoping review all categories of reports, ranging from individual institution annual reports to health organization reports, that would have provided a deeper understanding of the trend. However, this will necessitate translation in addition to other difficulties, as not all nations have such reports.

Thirdly, the USA was overrepresented in this review accounting for 47% of HIC studies. Although this highlights the ongoing issues in the USA with the opioid epidemic, it reduces the attempt to analyse trends of pharmaceutical poisoning in HICs in general. In addition, the reported trend may be understated due to the availability of panels of substance analysis in various nations. Despite this, the majority of poisoning cases are treated based on clinical judgment of the information acquired, and drug concentration monitoring is not always used to determine causality. Finally, where articles collected the data from poison databases, this often required voluntary reporting. Self-reported data has the potential risk of bias, thus, the accuracy of poison reports is unknown. Furthermore, data is also compiled from the volume of calls poison centres receive from physicians. However, many physicians are familiar with the diagnosis and management plan for often-occurring toxicities and so do not need to refer to the centres for advice. Thus, the available data is unlikely to comprehensively reflect the magnitude of the problem. 

## 5. Conclusions

This review is the first attempt to analyse the data available on pharmaceutical poisoning worldwide. Findings reveal that most drug toxicities are intentional in LMICs and accidental in HICs. Globally, the problem mostly lies with drugs acting on the nervous system, particularly analgesics, and medical outcomes from poisoning are generally worse in LMICs. Implementation of the suggested recommendations including the establishment of poison information centres worldwide, strengthening mental health resources, tightening medicine regulations, improving healthcare professional awareness surrounding drug toxicity and public health prevention campaigns would make a positive contribution towards alleviating the burden of these preventable injuries. Despite recognising the epidemiological patterns of poisoning, gaps in the literature were recognised calling for more robust analytical research. 

## Figures and Tables

**Table 1:** Search terms.

Poison Terms	Pharmaceutical Terms
Poison *	Pharmaceutical
Toxic	Medicine
Overdose	Drug
Intoxication	Opioid
Excessive	
Substance abuse	
Drug Misuse	

**Table 2:** List of all countries and their economic status where studies were conducted.

Low-Middle-Income Countries (n = 21)	High-Income Countries * (n = 58)
Algeria: 1 [38] Argentina: 1 [39] India: 2 [40,41] Iran: 9 [42,43,44,45,46,47,48,49] Jordan: 1 [50] Morocco: 1 [51] Romania: 1 [52] South Africa: 1 [11] Sri Lanka: 1 [53] Turkey: 3 [54,55]	Australia: 2 [56,57] Canada: 3 [58,59,60] Czech Republic: 1 [61] Denmark: 3 [62,63,64] Finland: 2 [65,66] France: 3 [67,68,69] Israel: 2 [70,71] Japan: 2 [72,73] New Zealand: 1 [74] Poland: 2 [75,76] Republic of Ireland: 1 [77] Switzerland: 4 [78,79,80] Saudi Arabia: 2 [81,82] Singapore: 1 [83] Taiwan: 1 [84] UK: 1 [85] USA: 27 [86,87,88,89,90,91,92,93]

* United Kingdom = UK; United States of America = USA.

**Table 3:** Drugs responsible for poisoning in LMICs and HICs grouped into the ATC 1st level categories *.

ATC 1st Level Classification	LMIC (n)	HIC (n)
A. Alimentary tract and metabolism	336	2721
B. Blood and blood forming organs	15	29
C. Cardiovascular system	193	2947
D. Dermatological	0	219
G. Genito urinary system and sex hormones	60	79
H. Systemic hormonal preparations, excluding sex hormones and insulins	66	30
J. Anti-infective for systemic use	185	635
L. Antineoplastic and immunomodulating agents	0	43
M. Musculo-skeletal system	496	37,736
N. Nervous System	3096	1,365,780
R. Respiratory system	478	2775
Combination of pharmaceuticals ingested	844	27,168

* Classification is according to the organ or system that the drugs therapeutically target.

**Table 4:** Drugs responsible for poisoning in LMIC and HIC studies categorised into the ATC 2nd level classification for drugs acting on the nervous system.

Nervous System	LMIC (n, %)	HIC (n, %)
Analgesics	1236 (39.9)	783,654 (57.3)
Antiepileptics	287 (9.27)	1194 (0.87)
Anti-Parkinson drugs	0 (0)	194 (0.01)
Psycholeptics	618 (20.0)	105,036 (7.69)
Psychoanaleptics	383 (12.4)	461,019 (33.8)
Drugs used in opioid dependence	572 (18.5)	14,683 (10.8)

## Data Availability

The data presented in this study are available on request from the corresponding author.

## Supplementary Materials

The following supporting information can be downloaded at: https://www.mdpi.com/article/10.3390/pharmacy11060184/s1, Table S1. Preferred Reporting Items for Systematic reviews and Meta-Analyses extension for Scoping Reviews (PRISMA-ScR) Checklist.

## Abbreviations

ADR	Adverse drug reaction
AKI	Acute kidney injury
ATC	Anatomic Therapeutic Chemical
BB	Beta blocker
CCB	Calcium channel blocker
CVS	Cardiovascular
CNS	Central nervous system
DDD	Defined Daily Dose
ED	Emergency departments
ENT	Ear, nose, and throat
GI	Gastrointestinal
HICs	High-income countries
ICU	Intensive care unit
LMICs	Low-middle-income countries
MI	Myocardial infarction
MALA	Metformin associated lactic acidosis
NSAID	Non-steroidal anti-inflammatory
OTC	Over the counter
POU	Pyrexia of unknown origin
PSS	Poisoning Severity Score
SSRI	Selective serotonin reuptake inhibitor
TCA	Tricyclic antidepressant
TB	Tuberculosis
UK	United Kingdom
USA	United States of America

## Appendix A. Search Strategy

1.	Poison*.mp.
2.	toxic.mp.
3.	overdose.mp or intoxication/
4.	excessive.mp.
5.	substance abuse/
6.	drug misuse/
7.	1 or 2 or 3 or 4 or 5 or 6
8.	pharmaceutical.mp.
9.	medicine/
10.	drug/
11.	opioid.mp.
12.	8 or 9 or 10 or 11
13.	7 and 12
14.	limit 13 to (English and yr = 2011–2020)

## Appendix B

**Table A1:** Characteristics and data extracted from included studies set in low-middle income countries.

Author (Year)	Study Design	Setting	Sample Size	Patient Demographics (Age (Years) * and Gender ^a^)	Reason for Exposure	Drug Responsible for Poisoning ^b^	Outcome from Exposure ^c^
Ghaemia et al. [42]	A prospective cross-sectional study at a tertiary toxicology centre in Northeast Iran	Northeast Iran	126	Ages 1–14: 126 Mean age: 2.8 M: 69 F: 57	Accidental: 126 Poor storage: 73 Parent mistakes: 14 Therapeutic error: 39	methadone: 65 buprenorphine: 6 Other opioids: 55	Symptoms: Drowsiness: 98 Apnoea: 35 Bradypnea: 47 Miosis: 87 Convulsions: 11 Hospitalised 126Deaths: 3
Hamedi et al. [49]	Cross-sectional study on children admitted to ED^b^ of Imam Reza Hospital. Data was collected from laboratory results and subjective data from parents’ responses.	Northeast Iran	79	(3 months–15 years) M: 45 F: 34	Accidental: 74 Ingested by child: 42 parents’ mistake: 28 Therapeutic error: 4 Intentional (suicide attempt): 1Unknown: 4	methadone: 79	Hospitalised: 79 Average length of stay: 31.77 h (2–87 h) Deaths: 2 (due to prolonged hypoxia and delay in hospitalisation)
Jabbehdari et al. [43]	Descriptive-sectional study on hospital admissions with methadone poisoning at Loghman-Hakim Hospital in the second half of 2012.	Iran	31	Mean age: 4.6 M: 16 F: 15	Accidental: 31	methadone: 31	Hospitalised: 31 Respiratory acidosis: 21 Leukocytosis: 17 Hyponatremia: 5 Prolonged QT interval: 7
Bilel et al. [38]	A retrospective descriptive study on poisonings received at the Oran University Hospital over 8 years using a pre-established information sheet on patient and circumstances of poisoning along with biological samples.	Algeria	400	4 m–5: 47 6–15: 48 16–25: 204 26–35: 73 >35: 26 (1 month–70 years) M: 100 F: 300	Accidental: 72 Intentional (suicide attempts): 328	paracetamol: 400	Hospitalised: 400 Average time hospitalised: 5 h–7 days Liver injury: 8 Death: 5
Kara et al. [124]	Retrospective study comprised records of patients admitted to ED of Konya Numune Hospital between 2009–2011.	Turkey	932	<15: 14 14–24: 617 25–34: 249 35–44: 86 45–54: 14 55–64: 14 >65: 22 M: 236 F: 696	Intentional (suicide attempts): 932	Antidepressant or antipsychotic: 162 Antiepileptic: 22 paracetamol: 71 NSAID: 59 GI drugs: 27 Antibiotic: 24 Antianemic: 15 Myorelaxant: 11 CVS drugs: 17 Anti-viral: 9 Contraceptive: 6 Respiratory drug: 6 Antidiabetic: 5 Antihistamine: 3 Co-ingestion: 249 Unknown: 246	Hospitalised: 932 Average length of stay: 24 h (24–168 h) Death: 1
Buffone et al. [39]	Descriptive, retrospective study based on the data collected from reviewing the medical records of patients 10–19 years at ED of Municipal Hospital of Bahia Blanca.	Argentina	72	Mean age: 16 (10–19) M: 20 F: 52	Intentional: 72 Family conflicts School conflicts Couple conflicts	Anxiolytics: 22 Analgesics: 10 Antihypertensives: 2 Antihistamines: 3 CNS stimulant: 2 Antidepressants: 2 Antibiotics: 1 Unknown: 30	Hospitalised: 72 Average length in hospital: 2.5 days Admitted to ICU: 1
Hocaoglu et al. [54]	Cross-sectional descriptive study reviewing theophylline exposure cases reported to Dokuz Eylul University Drug and Poison Information Centre (DPIC).	Turkey	354	<18: 146 >18: 206 M: 85 F: 257 Unknown: 12	Intentional: 291 Accidental: 46 Unknown: 17	theophylline: 354	Hospitalised: 354 Average length of stay: 17.1 h Death: 2
Mehrpour et al. [44]	Retrospective cross-sectional review based on hospital records of acute poisonings managed in ICU during a 7-year period in a single center in Birjand, Iran	Iran	267	20–35: 167 35–50: 46 50–65: 26 >65: 28 M: 173 F: 94	Accidental: 22 Intentional: 151 Suicide attempts: 102 Unknown: 49	paracetamol: 2 Benzodiazepine: 34 Antidepressant: 33 Antipsychotic: 20 Anticonvulsant: 4 Betablocker: 6 Opioids: 79 Co-poisoning: 8 Unknown: 81	ICU: 267 Death: 52
Azekour et al. [51]	Epidemiological retrospective study reviewing medicinal poisoning registered with the Provincial Delegation of Health in Errachidia between 2004–2016	Morocco	180	Mean age: 21 (2–75) M: 42 F: 131 Unknown: 7	Accidental: 101 Intentional: 72 Unknown: 7	alprazolam: 15 carbamazepine: 9 chlorpromazine: 9 trihexyphenidyl hydrochloride: 6 amitriptyline: 6 bromazepam: 6 lamotrigine: 6 paracetamol: 6 prazepam: 6 valproic acid: 6 bromazepam: 3 phenobarbital: 3 Contraceptives: 9 cyproheptadine: 3	Hospitalised: 132 Death: 3
Taheri et al. [125]	Descriptive analytical study performed from 2010–2012 in the poisoning emergency and clinical toxicology departments of Noor Hospital affiliated with Isfahan University of Medical Sciences.	Iran	385	Mean age: 32.1 (1–90) M: 294 F: 91	Intentional: 222 Accidental: 153	methadone: 385	Hospitalised: 385 Pulmonary oedema: 3 Aspiration pneumonia: 21 Death: 7
Weerasinghe et al. [53]	Retrospective analysis of self-harm cases. Data collected from primary and referral hospitals.	Sri Lanka	54	15–20: 32 21–25: 13 >26: 9 M: 2 F: 52	Intentional: 52 Accidental: 2	Contraceptives: 54	Hospitalised: 54 Average stay: 24 h
Bagherian Rad et al. [45]	Cross sectional retrospective study carried out on all patients referred to Loghman Hakim Hospital from 2011–2016.	Iran	229	Mean age: 24 (13–90) M: 77 F: 152	Intentional: 224 Unintentional: 5	NSAID: 217 Co-ingestion: 12	Hospitalised: 229 ICU: 8 Duration of hospital stay: <12 h: 6 12–24 h: 213 24–48: 7 >48: 3 Death: 1
Nagaralu et al. [40]	Retrospective review using data from ED at four tertiary care hospitals	India	708	1–20: 277 21–40: 277 41–60: 125 >61: 29 M: 407 F: 301	Intentional: 484 Accidentall: 149 Homicidal: 20 Unknown: 55	NSAID: 161 Antiepileptics: 117 Antidiabetics: 114 CVS drugs: 108 Antipsychotics: 78 Anxiolytics: 73 Anti-thyroids: 57	Hospitalised: 708 Death: 42
Anthony et al. [41]	Observational retrospective review using records from a tertiary care hospital over 15 months	India	91	Mean age: 28.1 M: 23 F: 68	Intentional: 72 Accidental: 19	Sedatives: 19 Antiepileptics: 19 Unknown: 53	ICU: 61 Average length in ICU: 3.94 days Deaths: 3
Shadnia et al. [46]	Retrospective cohort study using data from patients admitted to Loghman Hakim Hospital Poison Centre over 4-month period.	Iran	100	12–20: 38 21–30: 50 31–40: 8 41–50: 3 >50: 1 M: 82 F: 18	Intentional (suicide attempt): 93 Unknown: 7	tramadol: 100 Co-ingestion (paracetamol, benzodiazepines, clarithromycin and naltrexone): 15	Hospitalised: 100 Seizures: 100 Nausea: 13 Emesis: 10 Recurrent seizures: 7
Yehya et al. [50]	Retrospective descriptive study using data from PharmacyOne Poison call centre, 2014–2018	Jordan	900	<5: 306 6–10: 129 11–20: 32 21–50: 365 >50: 68 M: 473 F: 427	Intentional: 236 Suicidal: 228 Accidental: 596 Medical error: 68 Unknown: 68	Analgesics: 252 CVS: 76 CNS: 63 Antihistamines: 94 Vitamins and supplements: 15 Anti-diabetic: 30 Antibiotics: 70 Co-ingestion: 125 Unknown: 31	PSS No effect: 363 Mild effect: 355 Moderate: 150 Severe effect: 32
Van hoving et al. [11]	Retrospective review extracting data from Khayelitsha Hospital Emergency Care database	South Africa	192	<25: 91 25–35: 65 >35: 36 M: 60 F: 132	Intentional: 192	Analgesics: 68 CVS: 44 Antivirals: 28 Antibiotics: 27 Vitamins and Minerals: 24 Antihistamine: 15 Anticholinergic: 11 CNS: 11	Hospitalised: 192 ED: 154 ICU: 14 Referred to other hospital: 11 Death: 4
Hashemneiad et al. [47]	Cross sectional study using data from patients admitted with drug poisoning at Karaj Shariati Hospital over 1 year	Iran	172	Mean age 29.8 (12–80) M: 86 F: 86	Intentional: 172	Benzodiazepines: 50 Antipsychotic/antidepressant: 30 Opioids: 50 NSAID: 12 Methadone: 6 Unknown: 36	Hospitalised: 172 Seizures: 22 Death: 10
Yaylaci et al. [55]	Retrospective study of patients at follow-up admitted with intoxication to the ICU between 2009–2011	Turkey	153	17–25: 69 26–35: 51 36–45: 20 46–55: 7 >56: 6 Mean age: 29.4 M: 49 F: 104	Intentional (suicide attempt): 144 Accidental: 9	Multiple drugs: 47 Antidepressant or antipsychotic: 46 Analgesics: 20 Unknown 29	ICU: 153 Average length of stay: 2.4 days
Khodabandeh et al. [48]	Prospective cross-sectional study among acute drug poisoning patients at a single hospital over 1 year	Iran	410	<18: 42 18–24: 102 25–34: 154 35–44: 96 >45: 16 M: 249 F: 161	Accidental: 35 Intentional: 375 Suicide attempt: 71	Single drug: 222 Multiple: 133 Benzodiazepine: 103 Opioid: 98 Unknown: 209	Hospitalised: 410 Lung injury: 153
Sorodoc et al. [52]	Retrospective review using data from a single tertiary center from Iasi County, Romania	Romania	811	18–20: 132 21–30: 242 31–40: 201 41–50: 104 51–60: 72 61–70: 36 >70: 24 M: 272 F: 539	Accidental: 63 Intentional (Suicide attempt): 748	Benzodiazepines: 111 Antiepileptics: 101 Barbiturates: 69 CVS drugs: 48 NSAID: 24 Antidepressant: 22 TB drugs: 15 Antibiotics: 11 Unknown: 80 Antidiabetic: 7 Opioids: 5 Multiple drugs: 267 Unknown: 41	Hospitalised: 811 ICU: 162 Average length of hospital stays: 3.12 days Referred to psychiatric consultant: 666 Death: 2

* Where available, age ranges are displayed in brackets below the noted age categories and mean age of the sample size. ^a^ M:male; F: female. ^b^ NSAIDs: non-steroidal anti-inflammatory drugs; GI: Gastrointestinal; CVS: cardiovascular; CNS: central nervous system; TB: Tuberculosis. ^c^ ED: emergency departments; ICU: Intensive care unit.

**Table A2:** General characteristics of included studies set in high-income countries.

Author (Year)	Study Design	Setting ^a^	Sample Size	Patient Demographics (Age (Years) * and Gender ^b^)	Reason for Exposure	Drug Responsible for Poisoning ^c^	Outcome from Exposure ^d^
Jensen et al. [62]	A retrospective nationwide descriptive study using 2 databases; the Danish Poison and Information Centre (DPIC) and the State Serum Institute of Denmark. (SSI)	Denmark	1505	0–1: 52 2–5: 267 6–12: 54 13–16: 101 17–60: 900 >60: 82 Unknown: 49 M: 554 F: 907 Unknown: 44	Intentional: 1142 Suicide attempt: 514 Accidental: 350 Unknown: 71	promethazine: 556 cyclizine: 295 cetirizine: 232 loratadine: 132	Hospitalised: 456 Average length of stay: 1 day Max. length of stay: 14 days Deaths: 14
Martin et al. [86]	Retrospective review of all paediatric admission at Eastern Maine Medical Centre (EMMC) from 1999–2009.	USA	22	1–12: 16 13–17: 6 M: 10 F: 12	Accidental: 16 (All aged between 1–12) Intentional: 6 (All aged between 13–17) Drug source was family or friend for 82% of cases	methadone: 10 buprenorphine: 12	Hospitalisation: 22 Admitted to ward: 6 PICU: 16 Mean hospital stay: 2.3 days Range of stay: 1–7 days
Gregoriano et al. [94]	Retrospective analysis of reports to a National Poison Centre 1995–2013	Switzerland	40	0–18: 26 >18: 14 M: 21 F: 19	Children: Suicide attempt: 3, Accidental: 11 Adults: Suicide attempt: 22 Accidental: 4	Children: azathioprine: 9 6-mercaptopurine: 5 Adults: azathioprine: 26	Hospitalised: 40 Average length of stay: 2 days (1–11 days)
Martos et al. [78]	An observational study reported to national poison centre between 1995–2013	Switzerland	75	1–16: 30 17–83: 45 M: 24 F: 51	Accidental: 22 20 children 3 adults Intentional: 50 43 adults 8 children older than 12	tolperisone: 72 tolperisone and NSAIDS: 3	No effect: 6 adults 17 children Mild effect: 25 adults 10 children Moderate effect: 9 adults Severe effects: 5 adults 3 children
Cairns et al. [56]	Retrospective observational study. Data collected from NSW poisons information centre 2004–2014	Australia	1735	Mean age: 17 M: 820 F: 816 Unknown: 99	Intentional: 1735	dextroamphetamine: 575 methylphenidate: 1059 modafinil: 18 atomoxetine: 83	Hospitalised: 1594 Referred to toxicologist: 60
Alruwaili et al. [81]	Prospective, descriptive cross-sectional study looking at 2 paediatric ED in Riyadh over 2 years	Saudi Arabia	1035	<12: 1016 >12: 19 M: 528 F: 394 Unknown: 113	Unintentional: 906 Intentional: 22 Unknown: 104	Analgesics: 138 Anticholinergics: 57 CVS drugs: 56 Anti-diabetic: 52 Supplements: 42 Antipsychotics: 39 Antimicrobials: 3 salbutamol: 17 Birth control: 25	Hospitalised: 1035 Paediatric ward: 71 Paediatric ICU: 71
Eluri et al. [87]	Retrospective analysis of errors reported to USA poison control centre from 2000–2012	USA	533,763	0–5: 196,797 6–19: 101,365 20–49: 119,340 over 50: 90,179 Mean: 22.3 Age unknown: 26,082 M: 229,938 F: 302,919 Unknown: 906	Accidental: Therapeutic error: 484,360 >1 product with same ingredient: 21,361 Unknown: 28,042	paracetamol: 196,797 Opioids: 123,613 NSAIDs: 13,610	No effect: 80,236 Minor: 32,571 Moderate: 7077 Major effect: 1004 Death: 145 Unknown: 412,730
Ichikura et al. [72]	Cohort study from an ICU in Japan from 2006–2013	Japan	676	<19: 38 20–34: 328 35–49: 227 50–64: 47 >65: 36 M: 156 F: 520	Intentional: 676 Suicide attempt: 403	Benzodiazepine: 537 Antidepressants: 421 Barbiturates: 155 Antipsychotics: 279 Analgesics: 135 GI drugs: 77 Anti-Parkinson: 71 Antihistamines: 31 CVS drugs: 19 Anticonvulsants: 4 Other: 116	ICU: 676
Post et al. [88]	Retrospective analysis of calls to USA poison control centres (NPDS) from 2007–2016	USA	11,275	<6: 9709 6–12: 315 13–19: 1251 Mean age: 3.8 M: 5985 F: 5221 Unknown: 69	Accidental: 10,053 Intentional: 1001	buprenorphine: 11,275	Hospitalised: 8401 Deaths: 11
Kamour et al. [85]	Retrospective study using NPIS telephone enquires related to 4 NSAIDs between 2007–2013	UK	22,937	(14–98) M: 9596 F: 13,145	Intentional: 11,104 Drug misuse: 65 Accidental: 9826 Unknown: 602	mefenamic acid: 925 ibuprofen: 17,302 diclofenac: 3385 naproxen: 1325	CNS toxicity: 322 Seizures: 48 Confusion: 19 Anxiety: 9 Reduced consciousness: 163 Dizziness: 79 Agitation: 28
Tan et al. [83]	Retrospective review of paracetamol overdose presenting to a tertiary hospital in Singapore	Singapore	177	Mean age: 25 (21–36) M: 51 F: 126	Intentional: 136 Unintentional: 40 Intent unclear: 1	paracetamol: 177	Hospitalised: 177 Mean length of hospital stay: 3 days (1–28 days) Liver damage: 16 Liver failure: 2
Madadi et al. [58]	Retrospective study using the Office of the Chief Coroner of Ontario. All deaths coded drug-related were reviewed.	Canada	1359	Mean age: 44 Age range: 16–89 M: 867 F: 492	Accident: 924 Unknown: 221 Suicide: 214	Opioids: 1149 methadone: 210	Death: 1359
Austin et al. [89]	Population based study using North Carolina death certificate data to identify drug overdose decedents	USA	1221	Intentional mean age: 50.3 Unintentional mean age: 42.2 M: 711 F: 510	Intentional: 207 Accidental: 1014	oxycodone: 285 hydrocodone: 113 alprazolam: 278 clonazepam: 109 Antidepressants: 139	Death: 1221
Friedrich et al. [90]	Retrospective database analysis of NPDS from 2000–2015	USA	296,838	0–2: 50,399 2–6: 98,552 6–12: 17,900 12–18: 129,987 Gender not stated	Intentional: 142,482 Accidental: 154,356	alprazolam: 58,404 clonazepam: 53,836 lorazepam: 28,164 Another benzodiazepine: 156,434	Death: 253 Due to multiple drugs: 252
Torrents et al. [67]	A 6-year prospective national study. Patients identified using records reported to poison centre and contacted to complete survey	France	87	Mean age 2 (0.5–17 years) M: 40 F: 47	Accidental: 87	methadone: 87	Emergency unit: 42 Paediatric unit: 21 ICU: 13 Death: 5
Toce et al. [91]	Retrospective cohort study at a single paediatric tertiary care centre of children between 6 months and 7 years between 2006–2014	USA	88	Mean age: 2 (10 months–6.4 years) M: 45 F: 43	Accidental: 88	buprenorphine: 88	Hospitalised: 88 Respiratory depression: 73 Hypoxia: 25 Depressed mental status: 70 Agitation: 4 Misosis: 68 Emesis: 40
Gomes et al. [59]	Population-based cross-sectional study of patients admitted for acute care in hospitals across Canada due to prescribed opioids	Canada	2599	0–24: 332 25–34: 419 35–44: 373 45–64: 979 65+: 496 M: 1338 F: 1261	Accidental: 648 Intentional: 291 Unknown: 248	oxycodone: 294 fentanyl: 114 hydromorphone: 379 codeine: 199 morphine: 189 methadone: 205 buprenorphine: 22 tramadol: 27 Unknown: 12	Hospitalised: 2599
Shipton et al. [74]	Population based cohort study using records from the Coronial Services Office in Wellington from 2008–2012	New Zealand	325	0–9: 2 10–19: 7 20–29: 31 30–39: 71 40–49: 98 50–59: 70 60–69: 26 70–79: 9 >80: 11	Unintentional: 179 Intentional: 110 Unknown: 37	methadone: 99 Opioids: 226	Death: 325
Tadros et al. [92]	Retrospective study using data from the Nationwide ED Sample (NEDS) from 2006–2012	USA	21,928	Mean age: 9 (0–17) M: 10,528 F: 11,390	Intentional: 5316 Accidental: 13,524 Unknown: 2126	Opioids: 21,928	All ED visits Treated and released: 15,585 Admitted: 3464 Deaths: 11
Tadros et al. [93]	Retrospective cohort study utilising 2006–2011 data from the Nationwide ED Sample	USA	259,093	18–30: 60,709 31–40: 42,197 41–50: 56,900 51–60: 52,548 61–70: 26,146 71–80: 12,640 81–90: 6886 91–100: 1046 M: 123,398 F: 135,654	Unintentional: 138,603 Intentional: 68,641 Unknown: 51,849	Opioids: 259,093	All ED visits Treated & released: 108,504 Average charge treated in ED: $3,515.27 Admitted: 140,396 Average charge $27,491.87 for those admitted
Vakkalanka et al. [99]	Retrospective review of loperamide exposures reported to NPDS between 2010 and 2015.	USA	1736	<5: 5 6–12: 52 13–19: 331 20–39: 652 40–59: 355 >60: 257 Unknown: 84 M: 749 F: 980	Abuse: 228 Misuse: 569 Attempted suicide: 848 Other: 91	loperamide: 870 loperamide and co-ingestion with antihistamines, antipsychotics, antidepressants, alcohol, opioids, cough and cold remedies: 866	PSS No effect: 299 Minor effect: 387 Moderate effect: 384 Major effect: 126 Death: 15
Creswell et al. [126]	Cross sectional study. Data of children aged 0–19 exposed to opioids was collected using hospital admissions and Wisconsin Poison Control Centre (WPC)	USA	3320	0–5: 2019 6–12: 339 13–19: 962 M: 1634 F: 1681	Accidental: 2522 ADRi: 35 Therapeutic error: 613 Intentional: 748 Suicide attempt: 353 Unknown: 50	oxycodone: 361 tramadol: 352 Other prescription opioids: 2336 Opioid/paracetamol combination: 1679 Unknown: 428	ICU: 3320 Death: 3
Feingold et al. [70]	Retrospective study. Data was obtained from the National database on causes of death. Drug poisoning deaths were coded as opioid-related	Israel	875	15–24: 63 25–34: 142 35–44: 113 45–54: 78 55+: 26 M: 362 F: 60	Accidental: 9 Intentional: 4 Unknown: 409	Opioids: 875	Death: 875
Koskela et al. [65]	Retrospective study. Data was collected from Cause of Death Registry death certificates provided by Statistics Finland from 2007–2011.	Northern Finland	684	Urban: mean age: 47.5 (36–57) M: 292 F: 104 Rural: mean age: 52 (44–59) M: 226 F: 62	Urban: Intentional (suicide attempt): 82 Rural: Intentional (suicide attempt): 40	Urban: Benzodiazepine: 20 Antidepressants: 35 Antiepileptics: 33 paracetamol: 69 Insulin: 6 CVS acting drugs: 10 Rural: Benzodiazepine: 6 Antidepressants: 13 Antiepileptics: 19 paracetamol: 1 Insulin: 3 CVS acting drugs: 5	Death: 684
Tobaiqy et al. [82]	Retrospective study. Chart review of all acute paediatric poisoning incidence in ED at East Jeddah Hospital over 4-year period	Saudi Arabia	69	0–5: 41 6–11: 18 12–16: 10 M: 38 F: 31	Accidental: 46 Therapeutic errors Intentional: 5unknown: 18	Analgesics: 27 Anticonvulsant: 13 Antipsychotic: 9 CVS medicine: 3 Antihistamine: 1 Unknown: 16	Hospitalised: 69 Admitted to paediatric ward: 25 paediatric ICU: 8 Death: 1
Kriikku et al. [66]	Retrospective review of post-mortem toxicology cases positive for urinary buprenorphine between 2010–2014	Finland	775	Mean age: 31 M: 690 F: 85	Accidental: 463 Intentional parenteral instead of sublingual: 167 suicide: 90 other: 55	buprenorphine: 775	Death: 369
Thongprayoon et al. [127]	Retrospective review. Data extracted from the National Inpatient Sample (NIS) coded as ICD-9 diagnosis.	USA	13,805	<20: 3902 20–29: 3228 30–39: 1951 >40: 4710 M: 4810 F: 8994	Intentional (suicide attempt): 9029 Unknown: 4776	aspirin: 13,805	Hospitalised: 13,805 Mean hospital stay: 2 days >1 organ failure: 2761 Death: 132
Miller et al. [102]	Prospective cross-sectional study. Analysis of censuses of live emergency department and inpatient discharges for 11 USA states as well as Multiple Cause of Death census data between 2011–2012	USA	10,525	6–14: 26 15–20: 274 21–25: 571 26–30: 783 31–39: 1687 40–49: 2207 50–59: 2726 >60: 2251 M: 5660 F: 4865	Intentional (suicide attempt): 6716 Unknown: 3809	Antiemetic: 813 Antidepressant: 1714 Antidiabetic: 117 Antiepileptic: 688 Antiparkinsonian: 123 Barbiturate: 157 Benzodiazepine: 1551 Antispasmodic: 219 Opioid: 4386 NSAIDS: 105 Psychostimulant: 417 Antipsychotic: 653 Unknown: 4562	Death: 10,525
Manini et al. [128]	Prospective cohort study looking at two tertiary care hospitals over 12 months.	USA	274	Mean age: 40.3 M: 172 F: 102	Intentional: 217 Accidental: 57	Benzodiazepines: 59 Opioids: 54 Sympathomimetic: 50 paracetamol-containing: 50 Antidepressant: 40	Hospitalised: 274 MI injury: 12 Shock: 3 Dysrhythmia: 2 Cardiac arrest: 3 Death: 2
Lee et al. [57]	Retrospective review using data from calls to Victorian Poisons Information Centre (VPIC) over a 10-year period.	Australia	4412	5–14 years: 37 15–19 years: 325 20–74 years: 2152 >75 years: 7 unknown: 889 M: 1084 F: 2307	Accidental: 781 Therapeutic error: 517 ADR: 17 Intentional: 3631 Misuse: 221	quetiapine: 4412 co-ingested with: Antidepressant: 827 Benzodiazepine: 800 paracetamol: 296 Antiepileptics: 248 Antipsychotics other than quetiapine: 240 Opioids: 155 NSAIDs: 116 Hypnotics: 104 Mood stabiliser: 87	Hospitalised: 4412 Death: 1066
Vilay et al. [129]	Retrospective case-control study of exposures reported to the NPDS between 2001–2007	USA	9074	<18 years: 971 >18 years: 7811 Mean age: 44.3 M: 5468 F: 3591	Intentional: 5009 Accidental: 3152	Cardiac drugs: 506 paracetamol: 946 Salicylates: 450 lithium: 995 ibuprofen: 324 Stimulants: 841 Opioids: 620 Anti-infectives: 453 Antiepileptics: 284 Antipsychotics: 355 Anxiolytics: 286 Antihistamine: 473 Anticholinergics: 341 Diuretics: 1296 Other: 4270	ICU: 6326 Admitted to ward: 1387 Admitted to psychiatric facility: 137 Kidney injury: 9074
Wheatley et al. [130]	Retrospective review of poison centre records between 2001–2010	USA	162	Mean age: 27 M: 100 F: 62	Intentional: 49 Accidental: 113	Antiretrovirals: 162	ICU: 9 Coma: 1
Kominek et al. [75]	Retrospective analysis of patients hospitalised with paracetamol poisoning in a Paediatric Clinic between 2004–2012	Poland	44	Accidental mean age: 3 Intentional mean age: 15 (2–18) M: 7 F: 37	Intentional: 30 Accidental: 10 Dosing error: 4	paracetamol: 44	Hospitalised: 44
Haoka et al. [73]	Retrospective observational study analysisng medical records in a single tertiary hospital in Japan	Japan	145	65–74: 47 75–84: 61 85–100: 37 M: 54 F: 91	Accidental: 102 Intentional: 43	Benzodiazepine: 59 Antihypertensive: 18 Antipsychotic: 17 Antiarrhythmic: 14 theophylline: 8 Antidepressants: 7 Antihistamines: 3 Other hypnotics: 3 Opioid: 2 Other: 14	Hospital visits: 145 Outpatient: 80 Inpatient care: 65 Average length of hospital stay was 13.9 days (1–91 days)
Mroczkowska-Juchkiewicz et al. [76]	Retrospective evaluation of intentional poisoning cases in department of paediatrics, Childrens University Hospital in Lubin from 2007–2012	Poland	145	Mean age: 15.1 (12–15) M: 14 F: 131	Intentional: 145 reasons including psychiatric disorders, family conflicts, school conflicts, sexual assault, lack of self-acceptance from chronic disease	Co-ingestion: 46 CNS drugs: 52 paracetamol: 17 NSAIDs: 20 CVS drugs: 3 Antibiotics: 1 Chemotherapy: 3 Vitamins: 1 Laxatives: 2 Respiratory drugs: 1	Hospitalisation: 145 Acute hepatic failure: 2
Lasoff et al. [98]	Retrospective review using state-wide poison control system electronic database from 2002–2015	USA	224	Mean age: 41 (18–90) M: 103 F: 121	Intentional. Abuse: 8 Misuse: 71 Suicide: 83 Unknown: 62	loperamide: 224	Hospitalised: 64 Cardiotoxicity: 9 Deaths: 3
Feng et al. [131]	Cross-sectional study. Cases were identified from a database by ICD-9-CM diagnosis codes	USA	9647	<20: 691 20–34: 3361 35–54: 2947 55+: 2648 M: 4632 F: 5015	Accidental: 2305 ADR: 1663 Suicidal: 930	Opioids: 9647	Death: 53
Lavon et al. [71]	Prospective observational follow-up study of all medication errors outside healthcare facilities reported to IPIC	Israel	1381	<6 years: 814 >6 years: 397 unknown: 170 M: 673 F: 708	Accidental (therapeutic error): 1381	Analgesic: 378 Antibiotic: 169 ENT preparations: 96 Vitamins: 99 Cold and cough preparations: 88 Topical drugs: 65 CVS medications: 56 Sympathomimetic: 68 Antihistamines: 40 Hormones: 30 Other: 292	Tachycardia: 6 Hypotension: 4 Sedation: 18 Vomiting: 11 Abdominal pain: 10 Nausea: 6 Throat irritation: 3 Eye irritation: 14 Restlessness: 9 Weakness: 8 Hospitalised: 11
Stevens et al. [68]	Retrospective study analysing metformin poisoning reported to Western France PCC from 1999–2016	France	382	<15: 94 15–75: 221 >75: 61 Mean age 44.7 M: 174 F: 208	Accidental: 197 Intentional: 127 Therapeutic error: 58	metformin: 382	ICU admission: 90 AKI: 79 CV shock: 21 MALA: 63 Death: 21
Torrents et al. [69]	Retrospective descriptive study of cases of methadone exposure reported to French poison centres over a 7-year-period	France	1415	Mean age: 34 (10–74) M: 1001 F: 414	Misuse: 670 Suicide attempt: 584 Unintentional: 12 Medication errors: 140	methadone: 1415	Coma: 511 Seizure: 57 Somnolence: 949 Miosis: 598 GI effects: 140 Respiratory effects: 113 Death: 219
Zakharov et al. [61]	Retrospective review using the database of the Czech Toxicological Information Centre from 2007–2011	Czech Republic	2339	9–13: 316 14–18: 2023 M: 526 F: 1813	Intentional (suicide attempt): 2339	CNS drugs: 912 NSAIDs: 261 Respiratory: 119 CVS drugs: 64 Antibiotics: 37 Co-ingestions: 709 Unknown: 205	Medical care: 2339
Caupp et al. [100]	Retrospective review using Poison Control Centre Data in Ohio from 2002–2014	USA	619	10–14: 82 15–17: 224 18–24: 417 25–29: 344 M: 484 F: 423	Accidental: 97 Intentional: 504 Other/unknown: 37	tramadol: 125 paracetamol with hydrocodone: 112 paracetamol with oxycodone: 70 oxycodone: 35 buprenorphine: 30 co-codamol: 20 methadone: 18 morphine: 10 Antidiarrheals: 7 Others 125	PSS: No effect: 144 Minor: 319 Moderate: 287 Major: 83 Unknown: 213 Death: 9
Okic et al. [103]	Retrospective review of descents from forensic pathology in Kansas City autopsied between 2001–2011	USA	789	Mean age: 43 (2–92) M: 508 F: 281	Accident 332 Intentional (suicide attempt): 43 Unknown: 101	fentanyl: 180 methadone: 299 oxycodone: 310	Death: 789
Christenses et al. [63]	Retrospective review looking at enquires concerning CBBs reported to the Danish Poisons Information Centre (DPIC) from 2009–2015	Denmark	339	<16: 78 >16: 261 M: 146 F: 193	Intentional (suicide attempt): 156 Accidental: 183	CCB; amlodipine: 249 verapamil: 45 felodipine: 16 diltiazem: 16 Other: 13	Hospitalised: 275 Average length of stay: 1 day Death: 7
Truitt et al. [132]	Retrospective chart review of PCC charts by running a search on all calls received between 2007–2009	USA	436	<50: 85 >50: 351 Mean age: 65.1 (2–91) M: 152 F: 28	Accidental: 436	BB: 258 CCB: 178	Hospitalised: 32 Death: 1
Christensen et al. [64]	Retrospective study of drug poisoning cases reported to Danish Poison Information Centre (DPIC)	Denmark	239	0–6: 12 7–12: 6 13–25: 135 26–65: 82 65+: 4 M: 57 F: 182	Intentional (suicide attempt): 175 Accidental: 64	aripiprazole: 239 combined with: Antipsychotic: 78 Antidepressant: 72 Antiepileptic: 32 Benzodiazepine: 37 zolpidem or zopiclone: 15 paracetamol: 21	Hospital visits: 239 Sedation: 204 Tremor: 158 Survived: 239
King et al. [101]	Retrospective review of NPDS data from 2000–2014 to identify paediatric ADHD medication exposures	USA	156,365	0–5: 8891 6–12: 59,953 13–19: 37,521 M: 102,150 F: 53,970	Accidental: 128,119 Intentional: 23,034 ADR: 4040 Unknown: 1172	methylphenidate: 72,267 amphetamine: 69,642 atomoxetine: 13,303 modafinil: 1153	PSS: No effect: 47,549 Minor effect: 24,658 Moderate effect: 14,285 Major effect: 481 Death: 3
Vohra et al. [95]	Retrospective study of exposures using electronic health records and reports to NPDS (2004–2014)	USA	99	<4 years: 92 5–12 years: 7 Mean age: 1 M: 55 F: 44	Accidental: 88 Therapeutic error: 6 ADR: 4 Unknown: 1	benzocaine gel: 99	No effect: 74 Minor effect: 16 Moderate effect: 3 Major effect: 5
Lin et al. [84]	Retrospective evaluation of medical records of children under 18 who presented to the ED with pharmaceutical poisoning (2001–2008)	Taiwan	87	Mean age: 11.26 M: 39 F: 48	Accidental: 34 Intentional: 53	CNS agent: 42 Analgesic: 16 Respiratory: 6 CVS agent: 7 Vitamins: 6 Others: 10	Hospitalised: 87 POU observation: 60 Discharged from ED: 6 ICU: 4 Accidental average length of stay: 20.79 h Intentional length of stay: 37.74 h
Conner et al. [96]	Retrospective review of intentional self-poisoning (ISP) cases aged 13–65 treated at a USA University Medical Centre	USA	673	13–18: 218 19–65: 455 M: 237 F: 436	Intentional (suicide attempt): 673	Analgesic: 176 Anticholinergic: 142 Anticonvulsant: 46 Antidepressant: 209 Antipsychotic: 90 CVS drugs: 65 lithium: 20 Opioid: 68 Hypnotic: 182 Sympathomimetic: 66	PSS: Minor effect: 390 Moderate effect: 281 Deaths: 4
Piotrowska et al. [79]	Retrospective, observational study of patients presenting to ED of Bern University Hospital. Cases were identified using electronic database.	Switzerland	181	Mean age: 25 (16–85) M: 43 F: 138	Accidental: 38 Intentional: 143	paracetamol: 181	Hospitalised: 181 Average hospital length of stay: 2 days Deaths: 2
Patel et al. [97]	Retrospective, cross-sectional analysis using NPDS from 2010–2014 identifying patients <18 years with exposure to opioid.	USA	83,418	0–1: 5042 1–2: 32,204 3–5: 13,744 6–12: 8819 13–17: 23,245 M: 41,081 F: 42,022	Accidental: 61,206 Intentional: 20,064 ADR: 1088 Other: 227	Opioids: 83,418	PSS: No effect: 32,944 Minor: 32,443 Moderate: 7709 Major: 1368 Death: 111
Reichert et al. [80]	Retrospective review of acute single-agent exposures to pharmaceutical reported to Swiss Toxicological Information Centre (STIC) between 1997–2012	Switzerland	313	0–4: 14 5–9: 2 10–14: 12 15–19: 83 20–90: 193 M: 94 F: 219	Accidental: 42 Intentional: 266 Other: 5	Antidepressants: 136 Antipsychotics: 30 Antiepileptics: 17 Opioids: 15 methadone: 3 NSAIDs: 51 Other: 61	Seizures: 313
Sinyor et al. [60]	Retrospective review of drug induced suicides in Toronto using Coroner’s data	Canada	397	<20: 1 20–39: 109 40–59: 199 60–79: 70 >80: 17 M: 199 F: 197	Intentional (suicide attempt): 397	Opioids: 112 Anxiolytics: 105 OTC medicines: 85 TCA: 81 SSRI: 14	Death: 397
Glaizal et al. [133]	Retrospective review using results of a 2-year national survey by the toxicovigilance network (2008–2010)	USA	135	Mean age: 31 (13–58) M: 99 F: 36	Intentional (suicide attempt): 135	methadone: 135	ED: 85 ICU: 38 Death: 10
Cassidy et al. [77]	Prospective study over 3-years on medication errors reported to NPIC	Republic of Ireland	2348	<1: 291 1–4: 619 5–9: 185 10–17: 125 18–64: 450 64–80: 184 80+: 130 Unknown: 364 M: 1043 F: 1256	Accidental (medication error): 2348	Analgesics: 362 Antipsychotics: 163 Anti-epileptics: 90 NSAIDs: 206 Respiratory: 102 Antibiotics: 263 Contraceptives: 54 Opioids: 52 ACEIs: 50 Cough and cold: 149 Antihistamines: 136 Dermatologic: 55 Iron: 29 Vitamins: 20	PSS: No effect: 864 Symptomatic: 259 ED: 179
Calcaterra et al. [134]	Retrospective chart review of data from NPDS between 2001–2014	USA	188,452	Mean age: 31.5 M: 101,943 F: 85,580 Unknown: 929	Intentional: 188,452	Opioid: 57,338 Benzodiazepine: 95,353 Opioid and benzodiazepine: 20,555	Coma: 6264 Respiratory depression: 6766 Death: 124
Eigner et al. [135]	Retrospective review of overdose deaths using Indiana State Department of Health death certificates available through Allen County Coroner’s Office.	USA	418	15–24: 32 25–34: 86 35–44: 92 45–54: 123 55–64: 54 64–74: 10 75–85: 3 M: 249 F: 169	Accidental: 336 Intentional: 66 Unknown: 16	Opioids Benzodiazepines Antipsychotics Antidepressants Anticonvulsants Analgesics	Death: 418

* Where available, age ranges are displayed in brackets below the noted age categories and mean age of the sample size. ^a^ USA: United States of America; UK: United Kingdom. ^b^ M:male; F: female. ^c^ NSAIDS: non-steroidal anti-inflammatory drugs; CVS: cardiovascular; GI: gastrointestinal; CCB: calcium channel blocker; BB: beta blocker; CNS: central nervous system; OTC: over the counter; TCA: tricyclic antidepressant; SSRI: selective serotonin reuptake inhibitor; ACEI: angiotensin-converting enzyme inhibitor. ^d^ PICU: Paediatric intensive care unit; ICU: Intensive care unit; ED: emergency department; ADR: adverse drug reaction; MI: myocardial infarction; ENT: ear nose and throat; AKI: acute kidney injury; MALA: metformin associated lactic acidosis; POU: Pyrexia of unknown origin; PSS: Poisoning severity score.
